# Gender, school and academic year differences among Spanish university students at high-risk for developing an eating disorder: An epidemiologic study

**DOI:** 10.1186/1471-2458-8-102

**Published:** 2008-03-28

**Authors:** Ana R Sepulveda, Jose A Carrobles, Ana M Gandarillas

**Affiliations:** 1 School of Psychology, Autonomous University of Madrid, Spain; 2 Epidemiology Department, Public Health Institute. Region of Madrid, Spain

## Abstract

**Background:**

The aim of this study was to assess the magnitude of the university population at high-risk of developing an eating disorder and the prevalence of unhealthy eating attitudes and behaviours amongst groups at risk; gender, school or academic year differences were also explored.

**Methods:**

A cross-sectional study based on self-report was used to screen university students at high-risk for an eating disorder. The sample size was of 2551 university students enrolled in 13 schools between the ages of 18 and 26 years. The instruments included: a social-demographic questionnaire, the Eating Disorders Inventory (EDI), the Body Shape Questionnaire (BSQ), the Symptom Check List 90-R (SCL-90-R), and the Self-Esteem Scale (RSE). The sample design is a non-proportional stratified sample by academic year and school. The prevalence rate was estimated controlling academic year and school. Logistic regression analysis was used to investigate adjusted associations between gender, school and academic year.

**Results:**

Female students presented unhealthy weight-control behaviours as dieting, laxatives use or self-induced vomiting to lose weight than males. A total of 6% of the females had a BMI of 17.5 or less or 2.5% had amenorrhea for 3 or more months. In contrast, a higher proportion of males (11.6%) reported binge eating behaviour. The prevalence rate of students at high-risk for an eating disorder was 14.9% (11.6–18) for males and 20.8% (18.7–22.8) for females, according to an overall cut-off point on the EDI questionnaire. Prevalence rates presented statistically significant differences by gender (p < 0.001) but not by school or academic year.

**Conclusion:**

The prevalence of eating disorder risk in university students is high and is associated with unhealthy weight-control practices, similar results have been found in previous studies using cut-off points in questionnaires. These results may be taken into account to encourage early detection and a greater awareness for seeking treatment in order to improve the diagnosis, among students on university campuses.

## Background

Eating disorders are considered the third most prevalent chronic health condition among adolescent females [1]. Therefore, the epidemiology of eating disorders has focused mainly on adolescents. However, low prevalence of clinical cases among the general population constitutes the main difficulty of such study. Over the past decade, a refined methodology has been used to obtain a prevalence rate near to 4.5% in Western countries [2-5], including Spain [6-10], even though there may be a higher rate when considering all sub-clinical cases.

Eating disorders (ED), disturbed eating or body image dissatisfaction are not well documented in Spanish university populations, despite the fact that several studies have reported that these concerns and illnesses are present in this population [11-13]. Spanish university campuses do not have the facility to document reliably the number of cases accompanied by a major or minor mental disorder, or the needs and concerns related to student mental health. Nevertheless, there are sufficient results to indicate that university students may have a higher proportion of unhealthy eating behaviours and attitudes and may be considered a high-risk group [14-17].

A review of the existing literature reveals that there are several studies of college populations that observe the at-risk prevalence for eating disorders to be between 0.9–3% in males and 7.3–18% in females (however the overall average is around 11%). The majority of these studies estimated prevalence using specific self-report questionnaires in which a cut-off score was established, for example, the Eating Attitudes Test, EAT-40 [18] or EAT-26 [19] or on some scales from the Eating Disorder Inventory (EDI) [20] as Drive for Thinness (DT) or Body Dissatisfaction (BD) [21-23]. Furthermore, a cut-off point on the EDI total score has also been used as a screening tool, using a cut-off point of 50, 40 or 43 [7,11,24,25]. Other studies do not establish cut-off scores in the questionnaires and only describe eating disordered behaviours such as bingeing, vomiting or laxative misuse, and other symptoms of clinical relevance (e.g., restrictive dieting, weight and body image concerns). As these students fail to meet the diagnostic criteria [16,26-29], it is difficult to compare results between studies, in spite of the fact that those who do not meet the specific criteria for an eating disorder may nonetheless experience a significant amount of distress related to their eating-disordered behaviour [30]. In addition, several studies have demonstrated the predictive value of using self-report measures of eating habits as far as the risk to develop an eating disorder is concerned [14,16,31,32] and in spite of the limitations, it seems to reveal reliable indicators of unhealthy weight control practices and harmful attitudes among populations where boundaries of clinical relevance are still difficult to define with accuracy.

Recently, there have been a number of studies that have explored gender differences in eating disorders, while previously they have mainly focused on females [33-35]. However, a number of reports suggest that eating disorder psychopathology and psychiatric comorbidity in males and females are more similar than they are different [34]. As Carlat and Camargo [36] indicated that males account for 10% to 15% of all BN cases, with 0.2% of all adolescents meeting the full criteria of bulimia nervosa. Specifically, studies carried out among males in college populations showed that the frequency of eating disordered behaviours is also higher: 26% vomiting behaviour, use of laxatives, 41% binge [29]; 8% dieting, 10% bingeing and 2% laxatives [26]; 5% bingeing [11]; and 10% reported fasting, diet pills or use of laxatives or vomiting to lose weight [16]. This apparent contradiction suggests the need of further research in this area.

Additionally, the cut-off points on the questionnaires are established based on the female population, therefore they have not been validated for male samples and there exists no specific items to assess other unhealthy behaviours that might be associated specifically with males [36]. For example, Geist, Heinmaa, Katzman and Stephens [37] found differences in the type of body image preoccupations among men and women, where males are less concerned with exact weight or clothing size and are more concerned with achieving an idealised masculine shape. The scoring on the EDI revealed that males exhibited a statistically significant lower drive for thinness and body dissatisfaction than did females. Similarly, Joiner, Katz and Heatherton [38] found that the females scored highest on the drive for thinness scale, while on the contrary, males showed the most perfectionism and interpersonal distrust in a sample of adolescents with bulimic symptoms. The validity and discrimination of the EAT and EDI's subscales with cut-off scores has not been successful in screening the male sub-clinical population [39].

On the other hand, the college environment also promotes high stress and anxiety that may contribute to future patterns of eating problems, especially in students with competitive and perfectionism personalities [31,40-43]. Futch and colleagues [41] indicated that medical students scored significantly higher on the DT scale compared to students pursuing other avenues of study, such as history or sociology. This suggests that the prevalence of students at risk for an ED may increase with the competitiveness of the school or program.

This study was carried out to explore the prevalence of Spanish university students at high-risk for developing an eating disorder. The Eating Disorder Inventory (EDI-2) [44] was used as a screening instrument to detect the high-risk population based on previous results in a Spanish population. We acknowledge that the EDI is not a diagnostic instrument, however, it allows researchers to estimate the proportion of the population within the spectrum of disordered eating behaviours and psychopathological traits that are closer to the clinical population than the normal population. Specifically, the aims of our study were the following: (1) to examine disordered eating behaviour and psychopathological symptoms by gender, (2) to estimate the total prevalence of the university population at high-risk for developing an eating disorder by gender, academic year and school (3) to evaluate variables associated with the population at high-risk for an eating disorder. Specifically, it is hypothesized that students that reach the overall EDI cut-off point will be associated with (i) increased unhealthy weight-control practices, (ii) increased body image dissatisfaction, increased psychopathology and lower levels of self-esteem. Additionally, (iii) we expect differences by gender when you compare at high-risk prevalence rate, (iv) we expect differences by academic year when you compare at high-risk prevalence rate and (v) we expect differences by schools when you compare at high-risk prevalence rate.

## Methods

### Subjects and sampling method

The study was conducted with university students enrolled in the first and fourth academic year at the Autonomous University of Madrid, between October and April in the 2000–01 academic year. Of the 21 schools on the campus, 13 schools with the highest number of students enrolled were selected. A total of 10,150 students were targeted. Two schools had placements outside of the university in the fourth academic year, thus, students from third academic year were included. To achieve a representative sample of the university campus by academic year and school, the sample design was proportionally stratified according to academic year and school, assuming a 95% confidence interval and 0.05 of sampling error. A total of 4,682 students was identified as the desired sample size. The prevalence rate was estimated controlling academic year and school.

### Procedure

This study was carried out in several university schools of the Autonomous University of Madrid by the author and a team from the School of Psychology. The team consisted of 10 psychology students in their last academic year who were previously trained as interviewers. Once authorization was given by the University Dean to carry out the study, an invitation was sent by letter to each chosen school. Permission was given by the Deans of the different University Schools for the ED epidemiological study. We contacted personally by telephone or email the lecturers to explain the study. Permission was given by each lecturer to administer an anonymous and voluntary battery of questionnaires in his or her classroom. The students were not reimbursed for their time (45 minutes) completing the battery. Students were given the option of receiving the results of their assessment if they gave a telephone number and coded name.

### Measures

All participants completed the following questionnaires:

#### Demographic Questionnaire

This questionnaire collected demographic variables (age, weight and height self-reported, marital status, parental education, employment status, cohabitation, psychiatric history) and information on health habits (weight control compensatory strategies as dieting, vomiting or use of laxatives, regularity of the menstrual cycle, time invested in exercise and/or sport, alcohol and/or cigarette consumption). Body mass index (BMI = weight (kg)/height (m)^2^) was calculated based on self reported height and weight. According to DSM-IV [45], a body mass index less than or equal to 17.5 kg/m^2^, is considered as a diagnostic criteria for anorexia nervosa.

#### Eating Behaviour and Attitudes

The Eating Disorder Inventory (EDI-2) [46] consists of 91 items rated on a six-point scale (from 1 (never) to 6 (always)) that are divided into 11 subscales. The first 64 original items are grouped into 8 scales and additionally, 27 new items were added to form 3 more scales. It was designed for the assessment of attitudinal and behavioural dimensions relevant to anorexia and bulimia nervosa. The most extreme response is recoded as a 3, the immediately before is recoded as a 2, and the next response is recoded as a 1. The other three responses recodes as a 0. Scale scores are the sum of all items for each subscale. This questionnaire has good internal consistency between 0.84 and 0.92 for each scale. The version employed for this study was the Spanish version by Garner [44] which has also good psychometric properties. Higher scores indicate higher disordered eating attitudes and behaviours.

The findings of the two-stage epidemiologic study in a Spanish population sample by Gandarillas and colleagues (2003) [47] was taken into account, as these authors conducted a precise validation of EDI in a non-clinical adolescent population. In the first stage the questionnaire EDI was administered to a representative sample of 1534 female school students between 15 and 18 years old. In the second stage, all the students took part in a clinical interview. Alpha coefficients were 0.92 for the total scale and oscillated between 0.63 and 0.88 by subscales. The best cut-off point on the EDI total score (adding 8 original scales) was examined using receiver operating characteristic (ROC) curve analysis which calculated the probability of correct classification or prediction between clinical and non-clinical population. For a cut-off score > = 40 (obtained by 27.1% of the sample), the test presents a sensibility of 86.1% (69.7–94.8), specificity of 74.9% (72.1–77.5). For a cut-off score > = 50 (obtained by 17.4% of the sample), the test shows a sensibility of 72.2% (54.6–85.2), specificity of 84.5% (82.1–86.6). To discriminate male students at risk, an overall cut-off point of 40 was selected.

#### Body Image

The Body Shape Questionnaire (BSQ) [48] consists of a 34 item scale with scores between 1 (never) to 6 (always) for each item. It measures personal body dissatisfaction, fear of gaining weight and the desire to be thin. The Spanish version adapted by Raich and colleagues [49] was used which has an internal consistency of 0.97. A higher score indicates more body dissatisfaction.

#### Self-Esteem

Rosenberg's Self-Esteem Scale (RSE) [50] was used to assess the level of self-esteem. The RSE consists of 10 statements regarding a person's general beliefs about him/herself. Each item is measured on a four-point scale–from strongly agree (3) to strongly disagree (0). Five items are reverse scored–from strongly disagree (3) to strongly agree (0) so that in each case scores go from less to more self-esteem. The RSE has high reliability (Cronbach's alpha = 0.93) [51]. The Spanish version of the scale used for this study has good internal consistency with a coefficient alpha of 0.88 [52]. Lower scores indicate lower self-esteem.

#### General Psychopathology

The mental health of the sample was assessed using the Symptom Check List 90 Revised (SCL-90-R) [53], Spanish version by Gonzalez de Rivera and colleagues [54]. It consists of 90 questions that gauge nine symptomatic dimensions of psychopathology. The Global Severity Index (GSI) indicates the level of psychological distress for each individual. The internal consistency is between 0.81 and 0.90. Higher score means higher psychological distress.

### Definition of population at high-risk for an ED

The prevalence rate was estimated using the EDI questionnaire as a screening tool. For the current study, students who scored 40 or higher on the EDI total score in the screening were defined as a high-risk population of developing an eating disorder.

### Statistical analysis

The results were analysed using frequency distributions by gender. Student data were *t*-tested for continuous variables and chi-squared tests were used for each categorical variable by gender. Prevalence estimates of the high-risk population for ED and corresponding 95% confidence intervals (CIs) were calculated according to the academic year and school by gender, once the design was determined, according to a cut-off point on the EDI total score. A logistic regression analysis was used to investigate adjusted associations between school, academic year, gender while providing the proportion of the sample at high-risk for an eating disorder, odds ratios (OR) and confidence intervals. Statistical significance for gender and the high-risk population differences for categorical variables were calculated using the chi-squared test. A Pearson correlation coefficient, with the two-tailed test of significance, was used to assess relationships between scales. Mean scores in the BSQ, SCL-90-R and RSE scales were divided into quartiles according to high-risk or low-risk group and was calculated by gender. The highest quartile (Q3) indicated higher scoring in the other scales. All *p *values were two-tailed and statistical significance was set at p < 0.05. Data was analysed with the program Statistical Package for Social Sciences (SPSS 10.0).

## Results

### Response Rates

Of the 4,682 students that were calculated as the optimum sample size, 2,551 students participated in the epidemiology study; the response rate for student participation was 54.5%. The main reasons for of lack of participation were absenteeism and the teacher's non-presence during the data collection. A total of 2,386 students were included for the data analysis based on the number of returned valid questionnaires, of which 31.4% (n = 743) were men and 67.9% (n = 1620) were women. The percentage of invalid questionnaires was 5.4% (n = 140), including those that did not answer two or more questions, and 0.9% (n = 25) were not taken into account because they did not fit the age range.

The Medicine and Psychology Schools provided the highest response rates (100%) for the first year. Biology and Psychology Schools provided the highest response rates (79.8% and 63.7%, respectively) for the fourth year. The lowest response rate was provided by the Information and Technology (IT) School (32% for the first year and 34% for the fourth academic year). The age range was from 18 to 26 years old, of which 62.8% (n = 1,479) belonged to the first academic year and 29.8% (n = 709) belonged to the fourth, except for the Medicine and Teaching Schools, of which 7.4% belonged to the third academic year (n = 175) due to the fact that they had placements outside of the school in the fourth academic year. This group of students was coded as being in the fourth academic year for purposes of data analysis.

### Socio-Demographic Variables

The mean age of the first academic year was 19 (SD = 1.6) and the mean age of the students in the fourth academic year was 21.8 (SD = 1.6). Females were the majority in each group. Of all the students, 85.6% lived with both parents; 86% of the parents were married, 9% were divorced or separated and 4.6% were widowed. No gender differences were observed for the demographic variables. Gender differences were found for parental education, where a higher number of male students reported having fathers with a university degree (43.3% vs. 28.9%; χ^2 ^= 6.7, df = 2, p = 0.034).

Regarding the health habits of the students: 30% reported they did exercise, with the majority of men indicating they worked out at the gym (43.3%; 5 hours/week) and 50% of the women indicating they did aerobics (4 hours/week). Thirty-four per cent of students engaged in sporting activities; usually football for men (54%; 4.4 h/w) and swimming for women (43%; 3.4 h/w). There was a significant gender difference in terms of the number of hours dedicated to doing exercise or sport (t = 4, p < 0.001). Thirty percent of the students smoked and women smoked more frequently than men (34% vs. 22%; χ^2 ^= 33.6, df = 1, p < 0.001). Binge drinking was more frequent among men than among women (21.2% vs. 6.4%; χ^2 ^= 119, df = 3, p <0.001).

Help-seeking at a mental health service: 10% of the males and 12.5% of females reported having requested psychological or psychiatric services (χ^2 ^= 3.8, df = 1, p = 0.05); 32% of the sample did not specify the reason but those students that answered reported depression (19%) and anxiety (14.5%) as the most common problems among men and depression (17.4%), anxiety (14.5%) and eating disorders (12.5%) as the most common problems among women.

### Gender Differences in Psychopathological Symptoms and Eating Behaviour

Female students obtained higher drive for thinness (DT) and body dissatisfaction (BD) mean subscale scores than males (t = 11.0 and t = 15.0, p < 0.001, respectively), while the mean score in the bulimia (B) scale was greater for males (t = 2.3, p = 0.02). Excessive concern about shape and/or weight was assessed by the BSQ, for which the females had significantly higher means compared with the males (mean = 73.1 (28) vs. 52.2 (18.5), respectively; t = 17.5, p < 0.001). There were significant mean differences by gender in the SCL-90-R scales (p < 0.001). Men had higher scores for hostility, paranoia and psychosis and females had higher scores for somatisation, interpersonal sensitivity, depression and anxiety. The GSI score was greater for females than for males (mean = 0.72 (0.5) vs. 0.66 (0.5); t = 2.3, p < 0.023). There were no significant differences by gender in the RSE (mean = 32 (5); t = 0.7, p = 0.50).

Based on self-reported height and weight, the mean BMI for men was 22.9 (SD = 2.6) and 20.6 for women (SD = 2.4). The mean desired BMI for men was 22.8 (SD = 1.8) and 19.8 for women (SD = 1.5). Difference by gender was statistically significant (t = 20.0, p < 0.001). 1.3% (n = 9) of the males and 6% (n = 83) of the females reporting a BMI of 17.5 or less. Dieting (9.3% vs. 18.6%; χ^2 ^= 33.1, p < 0.05), laxatives use (2.9% vs. 5.5%; χ^2 ^= 7.4, p < 0.05) and vomit to reduce weight (2.6% vs. 4.6%; χ^2 ^= 2.8, p < 0.05) was most prevalent for females. Amenorrhea was reported by 2.5% (n = 42) of the female sample where only 7 of the females self-reported an ED. Only binge eating was higher in the male sample (11.6% vs. 7.2%, χ^2 ^= 4.5, df = 1, p < 0.05). There were significant differences by gender for all of the behaviours described above.

### Prevalence of University Students at High-Risk for ED by Gender, Academic Year and School

**Figure 1:**
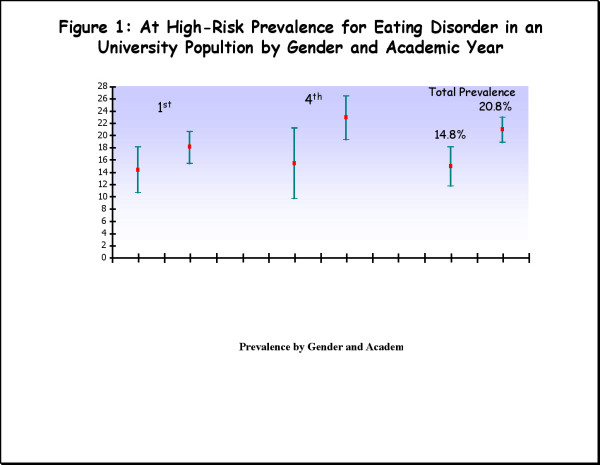
At high-risk prevalence rate for an ED according by gender and academic year (95% C.I.).

One percent of the total sample (n = 27) reported that they were receiving or had received treatment for an eating disorder. The prevalence of university students at high-risk for developing an eating disorder was 17.6% (19.6–16.1), 14.8% (18-11.6) for men and 20.8% (22.8-18.7) for women. There were significant differences by gender are shown in Figure 1 (χ^2 ^= 21.0, df = 1, p < 0.001).

Regarding academic year and gender, 14.3% (18-10.5) of the males and 18% (20.4-15.4) of the females in the first academic year were at greater risk for ED. In the fourth academic year, 15.4% (21.1-9.6) of the males and 22.8% (26.4-19.2) of the females were at greater-risk of developing an ED. There were no significant differences by academic year (χ^2 ^= 2.6, df = 1, p = 0.10).

**Figure 2:**
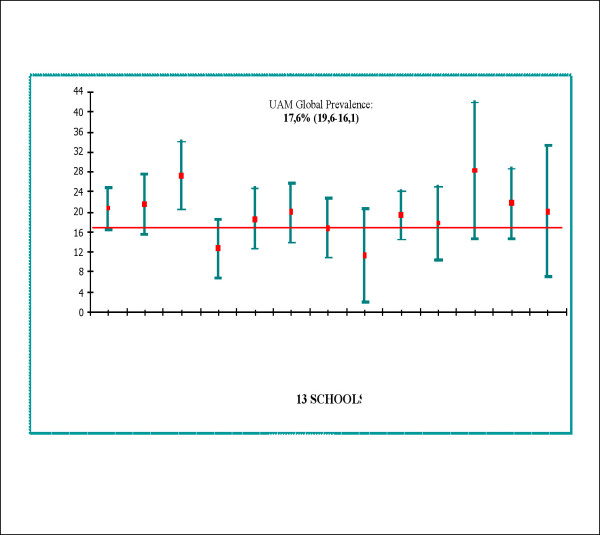
At high-risk prevalence rate for an ED according by school (95% C.I.).

The prevalence of university students at high-risk for developing an eating disorder by school varied from 11.2% to 28.2% and is presented in Figure 2. The lowest prevalence rates were found in the IT School, at 11.2% (20.6-2), and the Economics School, at 12.8% (18.6-6.7). The highest prevalence rates were found in the Hispanic Literature School, at 28.2% (41.7-14.6), and the Law School, at 27.2% (34-20.3). However, there were no significant differences by school and gender (χ^2 ^= 22.2, df = 12, p = 0.07). In each school, females had the highest prevalence rates.

A logistic regression was performed with an EDI cut-off point as the dependent variable and school, academic year and gender as independent variables, and their various interactions as predictors. Results showed that school (OR = 1.07; 95% CI: 0.94 – 1.21; p = 0.30) and academic year (OR = 1.02; 95% CI: 0.89 – 1.15; p = 0.82) were not associated with a significantly higher risk of developing an ED, while gender was (OR = 1.16; 95% CI: 1.08 – 1.24; p < 0.001). The interaction effect between the three variables did not emerge as a significant predictor of developing an ED (OR = 0.99; 95% CI: 0.98 – 1.0; p = 0.12), while gender was the only factor associated with the risk of developing an ED.

### Gender Differences for Population at High-Risk for an ED Related to Psychopathology Symptoms

The percentages of unhealthy eating attitudes and behaviours amongst the high-risk population compared with the low-risk population by gender are shown in Table 1. All of these disordered eating patterns were more frequent amongst the high-risk group, except for the body mass index (BMI), where a greater number of women had a BMI of 17.5 or less (3.1% vs. 6.7%, χ^2 ^= 5.4, df = 1, p = 0.023). Both female groups presented statistically significant differences in unhealthy weight-control practices compared with the male groups. Although, binge eating behaviour was more frequent among male students.

**Table 1:** Comparing disordered eating between low-risk and high-risk groups for an ED by gender

**Unhealthy Eating Behaviours**	**ED Low-Risk**		**ED High-Risk**		
	Males	Females	Males	Females	
	N = 620	N = 1238	N = 99	N = 350	**p**
*Behaviours*					
BMI < = 17.5	1.7%	**9.9%**	2.2%	**3.2%^a^**	**0.001^a^** 0.001*
I have gone on diets	7%	**13%**	22.4%	**38%^a^**	**0.001^a^** 0.001*
I have used laxatives to get thin	1.8%	**2.7%**	10.7%	**14.6%^a^**	**0.001^a^** 0.001*
Amenorrhea 3 months or more	--	**1.5%**	--	**6.2%^a^**	**0.001^a^** 0.001*
I frequently have binging episodes where I can not stop eating	7%	**4%**	26.3%	**19.7%**	**0.001^a^** 0.001*
I have vomited to get thinner	1.8%	**1.4%**	9.6%	**16%**	**0.001^a^** 0.000*
*Eating Attitudes*					
Thinking about dieting	6%	**21.2%**	29.3%	**70.8%^a^**	**0.0001^a^** 0.001*
I am afraid to gain weight	7.9%	**25.7%**	43.4%	**74%^a^**	**0.001^a^** 0.001*
I have a great desire to be thin	7%	**22.3%**	27.7%	**74%^a^**	**0.001^a^** 0.001*

^a^p = between males and females of high-risk vs. low-risk.

*p = between males high-risk vs. low-risk/females high-risk vs. low-risk

Gender differences for high scores on EDI subscales were explored. Men scored significantly higher on the ineffectiveness (t = 2.5, p = 0.02), perfectionism (t = 3.1, p = 0.01), interpersonal distrust (t = 4.6, p = 0.01), and maturity fear subscales (t = 3.0, p = 0.01) compared with females at high-risk for ED. However, there were no significant gender differences in the bulimia and interoceptive awareness scores (t = 1.2, p = 0.3 and t = 1.1, p = 0.08). Females, by contrast, scored significantly higher in the drive for thinness and body dissatisfaction when compared with high-risk males (t = -7.9 and t = -9.9, p < 0.05, respectively).

A partial correlation was estimated for the overall score of EDI and each scale after controlling for gender. A significant positive correlation coefficient was found for the overall score of EDI and BSQ (r = 0.71, p < 0.001) and for the psychopathology index (r = 0.59, p < 0.001). In contrast, a statistically significant inverse association was found between the overall score of self-esteem and the overall score of EDI, BSQ and Global Severity Index (GSI) (r = -0.53, p < 0.001, r = -0.39 and r = -0.56, p < 0.001, respectively).

**Figure 3:**
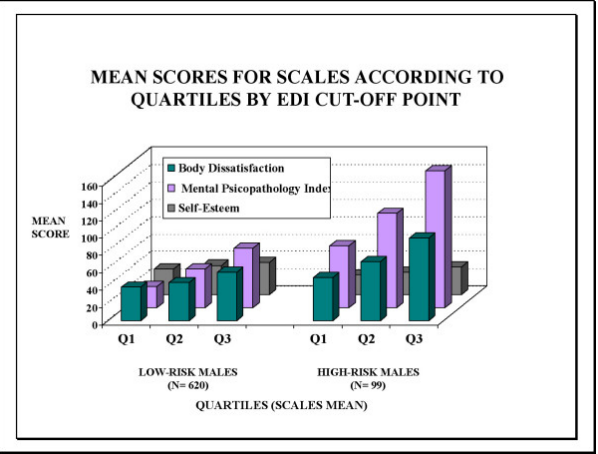
**Prevalence of the scales' scorings according to students at risk group IN MALES.** Mean scores for scales according to quartiles by EDI cut-off point.

**Figure 4:**
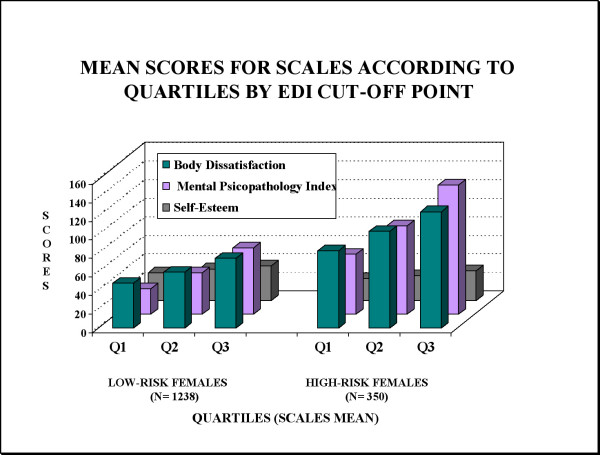
**Prevalence of the scales' scorings according to student at risk group IN FEMALES.** Mean scores for scales according to quartiles by EDI cut-off point.

Figures 3 and 4 show the mean scores for the BSQ, SCL-90-R and RSE scales according to quartiles based on EDI scores in males and females at high-risk or low-risk for an ED (Q1: lowest scoring, Q3: highest scoring). Female and male students at high-risk were associated with higher levels of body dissatisfaction by BSQ and psychological distress (GSI) compared to students at low-risk for an ED. Gender differences were also found in the high-risk student group, where there was a trend toward higher scores on the measure of psychological distress (GSI) in males at high-risk, while their female counterparts showed a similar tendency, with higher scores on the BSQ scale.

## Discussion

The first aim of this study was to determine the impact of gender-related differences in disordered eating behaviour and psychopathological symptoms among university students. Results from female students indicate greater need for concern related to eating behaviour, body dissatisfaction and psychological distress compared to male students. In addition, female students presented unhealthy weight-control behaviours as dieting, laxatives use or self-induced vomiting to lose weight than males. A total of 6% of the females had a BMI of 17.5 or less and 2.5% had amenorrhea for 3 or more months, meeting two of the diagnostic criteria for anorexia nervosa. In contrast, the bulimia score was greater for males and a higher proportion of males (11.6%) reported binge eating behaviour. Therefore, exploring gender differences was relevant in understanding the prevalence of unhealthy eating behaviours and attitudes amongst university students, according to previous studies [11,16,17].

The second aim was to estimate the prevalence of a university student population at high-risk of developing an eating disorder using a large university representative sample by gender, academic year and school. An overall cut-off point of 40 on the EDI questionnaire was used to determine that 17.6% (19.6-16.1) of university students can be considered to be at risk of developing an eating disorder. Two main factors were taken into account, the decision to use an overall score as the screening point: a) the cut-off points given on the DT or B scales are not sensitive enough to detect ED symptoms in male samples and b) the two-stage epidemiological study using the EDI questionnaire among Spanish adolescents. Specifically, the previous validation study of the EDI [47] was performed in a representative female adolescent population where the sample had previously participated in a clinical interview. Using a cut-off score of 40, the authors observed that 27.1% of the sample was at high-risk of developing an ED which means that 31 clinical cases were detected from 42 cases. Two Spanish studies have used the global score of EDI (> = 50) to screen the at-risk population for ED. The first study by Morande and colleagues [7] found that 12% of males and 31% of females were at risk in an adolescent population. The second study by Lameiras and her team [11] reported that 6.7% of men and 6% of women were at risk of developing an ED in a university population. In addition, Machado and colleagues [24] validated the Portuguese version of the EDI and used a cut-off point of 43 to screen female college students [25]. The authors reported that 18.4% of the females reached this cut-off score, which is similar to the results of the current study.

In 1996, Becker and collaborators [55] carried out the first National Eating Disorders Program (NEDSP), which was conducted at more than 400 college campuses with 9,069 participants to detect disordered eating behaviors and provide secondary prevention. Although the overall prevalence of clinical individuals was unknown, nearly three-quarters of the sample received a recommendation for further clinical evaluation. A follow-up random sample was conducted 2 years later (N = 289), and it was found that nearly one half had made a first appointment with a counsellor for a clinical evaluation and 40% of those did seek treatment for ED. The prevalence of disordered eating was higher than expected, indicating that ED symptomatology was widespread amongst university students. Thus, it is crucial to identify the high-risk subpopulation and encourage greater awareness of treatment availability [16,55].

Statistically significant differences for gender were found among proportions of students at high risk for an ED, specifically 14.8% (18-11.6) for men and 20.8% (22.8-18.7) for women. This gender difference is apparently small compared with other studies, where the at-risk prevalence in males is lower (e.g., 2.2% in Gandarillas et al., [56]; 3,3% in Ruiz et al., [10]). This may be due to the fact that cut-off points for the DT, B or BD scales and cut-off point on the EAT were used, and that males apparently fell into the high-risk category due to other factors. In our sample, male students at high-risk also scored higher on the five scales related to psychopathological eating disorder traits, such as perfectionism or interpersonal distrust. This may point to the development of general global vulnerability for health more than scales related directly to weight and body shape attitudes and behaviours, which may be more closely related to a trend of vulnerability in women. However, students of both genders exhibited lower self-esteem, which supports this pattern of vulnerability.

The third aim of this study was to estimate the unhealthy eating behaviours that were associated with a population at high risk for developing an ED. The population at high risk for an ED also exhibited more frequent weight- or shape-control attitudes and behaviours (dieting, use of laxatives, vomiting, etc.) than the population considered low risk. For example, several studies found that males do not often engage in vomiting to control weight and/or their figure [26] but do engage in other at-risk behaviours, such as steroid or hormone abuse [57]. Males in the high-risk group reported more alcohol use and high-risk females reported smoking more cigarettes than the low-risk females. A higher percentage of males and females in the high-risk group indicated requesting psychological or psychiatric services at some point. Neumark-Sztainer and her team [58] found that adolescents with eating disorders indicated increased smoking, binge drinking, illegal substance abuse, and suicide attempt rates. The data of this study confirm the observation by Fisher et al. [59] that unhealthy behaviours are grouped in vulnerable adolescents, with unhealthy eating attitudes frequently a part of this aggregation.

### Limitations of the Study

Several factors must be taken into account when observing these prevalence rates. Despite the sample size, sampling type and wide spectrum of assessed behaviours, there are several limitations. Firstly, the study was a cross-sectional study and thus, we are not able to infer causality. Another limitation is the use of self-report questionnaires to collect data, as this method relies upon the honesty of the students. To mitigate this problem, questionnaires were conducted anonymously; however it is possible that biased answers were collected, in which case the prevalence rate may have been underestimated. The response rate for student participation was considered satisfactory, even though it was lower than that of other studies carried out in a university sample (66% in Forman-Hoffman [16] or 64% in Futch and colleagues [41]) but higher than the 37% response rate in the study conducted by Anstine and colleagues [21]. In this case, the result is due to the high absenteeism rate at public universities, where it is estimated that approximately 40% of the students do not attend any of the lectures, and 10% stop attending lectures after one month after starting the course. Attending lectures is voluntary and depends on the particular lectures and lecturers. Students were not notified of the questionnaire session before the day of collection and the presence of the lecturer during data collection was also associated with a higher rate of participation.

Finally, another limitation of the study is related to the chosen definition of 'population at high-risk for an eating disorder'. Bjomelv, Mykletun and Dahl [60] state that the various definitions of an "eating problem" have a low degree of correlation and lead to variable prevalence. In our study, students considered as at high-risk for an ED also presented higher prevalence of eating problems in females, although the prevalence among males was also considerable. Nevertheless, clinical interviews were not performed and consequently the accuracy of this prevalence cannot be assumed.

The similarities of core eating disorder psychopathology and comorbid illness in male and female patients encourage the continued use of similar detection with both groups [61]. Nevertheless, precise items in questionnaires to assess unhealthy behaviours that might be specific within male samples need to be developed [36].

Further research is necessary to examine the possible relationship between demanding environments and psychosomatic vulnerability, especially amongst university students. Health programmes have been recommended for educational services on university campuses [55]. In the light of this recommendation, our team has since carried out a healthy habits programme based on the improvement of body image and self-esteem with a university sample where positive results were obtained among female populations [56,62]. It is hoped that other such efforts will soon be attempted in an effort to alleviate potential risk factors and unhealthy behaviours and attitudes.

## Conclusion

The results of this study suggest that the prevalence of university population at high-risk of developing an eating disorders is high. A significant difference in prevalence rate has been found by gender but not by school or academic year. Unhealthy control-weight behaviours are associated to these vulnerable students, mostly amongst females, with psychopathological symptoms presented as part of this aggregation.

Specifically, female students presented more unhealthy weight-control behaviours to lose weight than males. A total of 6% of the females met two of the diagnostic criteria for anorexia nervosa. In contrast, the bulimia score was greater for males and a higher proportion of males (11.6%) reported binge eating behaviour.

Assuming the prevalence of students at high-risk for an ED and from a preventive point of view, early detection of a situation that poses risks and a better tailoring of treatment to that situation is essential in order to improve the diagnosis, especially among male populations.

## Competing interests

The author(s) declare that they have no competing interests.

## Authors' contributions

ARS conceived and designed the study, oversaw all stages of data collection and performed the statistical analysis and drafted the manuscript. JC coordinated all stages of the study, gave feedback on design and reviewed the manuscript. AG revised the data analysis, interpretation of results and reviewed the manuscript. All authors read and approved the final manuscript.

## Pre-publication history

The pre-publication history for this paper can be accessed here:


